# Prevalence of equine obesity in Sweden assessed from official animal welfare control data

**DOI:** 10.1186/1751-0147-57-S1-O7

**Published:** 2015-09-25

**Authors:** Peta Hitchens, Jan Hultgren, Jenny Frössling, Ulf Emanuelson, Linda Keeling

**Affiliations:** 1 Department of Animal Enviroment and Health, Swedish University of Agricultural Sciences, Uppsala, Sweden; 2 National Veterinary Institute, Uppsala, Sweden; 3 Department of Clinical Sciences, Swedish University of Agricultural Sciences, Uppsala, Sweden

## Introduction

Swedish animal welfare legislation states that horses should be fed so that they do not become over- or underweight.

## Objective

To estimate the prevalence of equine obesity in Sweden using information collected through official animal welfare control.

## Methods

The horse checklist for official animal welfare control contains 45 checkpoints (CP) of which CP8 pertains to the horses' body condition. For the years 2010-2013, prevalence of non-compliance with CP8 and 95% confidence intervals (CI) were estimated using the exact binomial distribution. Associations with risk factors were estimated using logistic regression and expressed as odds ratios (OR).

## Results

Of 7,877 premises with registered horses that controlled for CP8, a total of 63 premises had non-compliant inspections due to overweight or obese horses (0.80%; CI 0.62, 1.02%) and 861 were due to underweight or emaciated horses (10.93%; CI 10.25, 11.64%). In multivariable analysis, overweight or obese horses had greater odds of being observed in summer when compared to winter (OR 2.33; CI 1.06, 5.13; p=0.035), and when cared for by inexperienced or less knowledgeable personnel, as indicated by non-compliance with CP's on daily maintenance and supervision requirements, appropriate treatment of sick or injured horses, and/or feeding a balanced high-quality diet (OR 7.59; CI 4.74, 12.14; p<0.001).

## Conclusions

Official animal welfare control data may be used to monitor the prevalence of equine obesity in Sweden, although observation of underweight horses is more common. Strategies to reduce the prevalence of obesity should focus on education about equine care and nutrition, especially summer grazing.

